# Dual-energy computed tomography using a gantry-based preclinical cone-beam microcomputed tomography scanner

**DOI:** 10.1117/1.JMI.5.3.033503

**Published:** 2018-08-21

**Authors:** Justin J. Tse, Joy Dunmore-Buyze, Maria Drangova, David W. Holdsworth

**Affiliations:** a Western University, Bone and Joint Institute, Imaging Research Laboratories, Robarts Research Institute, London, Ontario, Canada; b Western University, Bone and Joint Institute, Departments of Medical Biophysics and Medical Imaging, London, Ontario, Canada; c Western University, Bone and Joint Institute, Department of Surgery, London, Ontario, Canada

**Keywords:** dual-energy computed tomography, x-ray filtration, resin filters, erbium, x-ray spectra, image co-registration

## Abstract

Dual-energy microcomputed tomography (DECT) can provide quantitative information about specific materials of interest, facilitating automated segmentation, and visualization of complex three-dimensional tissues. It is possible to implement DECT on currently available preclinical gantry-based cone-beam micro-CT scanners; however, optimal decomposition image quality requires customized spectral shaping (through added filtration), optimized acquisition protocols, and elimination of misregistration artifacts. We present a method for the fabrication of customized x-ray filters—in both shape and elemental composition—needed for spectral shaping. Fiducial markers, integrated within the sample holder, were used to ensure accurate co-registration between sequential low- and high-energy image volumes. The entire acquisition process was automated through the use of a motorized filter-exchange mechanism. We describe the design, implementation, and evaluation of a DECT system on a gantry-based-preclinical cone-beam micro-CT scanner.

## 1 Introduction

Microcomputed tomography (micro-CT) is widely utilized in biological studies for its capability of providing high-resolution images of contrasting tissues. Attenuation within micro-CT images is dependent on the materials’ effective atomic number (i.e., Z, electron density), where higher Z materials (i.e., higher electron density) will exhibit higher x-ray attenuation due to photoelectric absorption.^1^^,^^2^ For biological samples, bone can be easily visually separated from surrounding soft tissues (i.e., muscle, fat, etc.) due to its composition. However, discrimination between soft tissues is difficult, due to their relatively similar electron densities.^3^ To enhance the visualization of soft tissues, tissue-specific exogeneous contrast agents are commonly required.^4^^,^^5^ Vascular contrast agents (i.e., angiography) are routinely used to facilitate the visualization and distinction of perfused vasculature from surrounding noncontrast-enhanced soft tissues.^6^

Clinical angiography typically employs the use of iodine-based agents.^7^^–^^9^ X-ray images of perfused vasculature are markedly different—due to the increased contrast—from surrounding soft tissue, allowing for the automatic segmentation of contrasted-vessels based on grayscale values alone.^10^ However, due to the dilution of injected iodine-based contrast agents and its diffusion within the blood stream, the contrast enhancement of vessels is often masked by nearby dense bone. For *in vitro* and *ex vivo* applications, more x-ray attenuating vascular agents such as lead-based silicone elastomers (Microfil MV-122, Flowtech, Inc., Massachusetts, USA) can be utilized.^11^ Nonetheless, the mean CT signal level of perfused vessels appears similar to cortical bone, hindering their automatic separation based on grayscale values.^5^^,^^12^

Dual-energy microcomputed tomography (DECT) is an x-ray imaging technique that can facilitate the automatic decomposition and segmentation of materials based on their elemental composition. Dual-energy CT is used clinically for angiography^13^ and kidney stone identification.^14^ As the name implies, DECT involves scanning a sample at two different energy spectra—achieved through differing acquisition protocols. Each material exhibits a unique elemental x-ray attenuation signature; thus, within composite samples, DECT decomposes each material based on their differential contrast at two different x-ray energies. The performance and image decomposition of DECT can be further improved if the dual-energy spectra are tailored to a material’s absorption K-edge.

Every material possesses a unique absorption K-edge energy (i.e., the x-ray energy required to eject an inner K-shell electron), which leads to a significant increase in x-ray attenuation when x-ray energies are above the K-edge energy. Dual-energy CT performed with mean spectral x-ray energies above and below the K-edge of interest takes advantage of this increased attenuation to improve the decomposition of materials of interest. There are multiple avenues to performing DECT, whether it is through fast kV-switching,^15^^,^^16^ dual-source,^15^ or dual-detector CT^17^ scanners. However, the large installed base of conventional preclinical micro-CT scanners are limited to scanning at a single x-ray energy at a time. The inherently polychromatic nature (i.e., implement with a broad x-ray spectrum) of these micro-CT scanners requires the careful selection of dual-energy acquisition protocols, as spectral overlap may reduce the effectiveness of DECT decompositions.

Spectral separation between the low- and high-energy images can be achieved through spectral shaping with differential added filtration to optimize the performance of DECT.^18^^–^^21^ This has been successfully shown in previous research,^5^ where copper and lead foils were utilized to facilitate the necessary spectral separation, resulting in the DECT decomposition of a rat hindlimb (perfused with a lead-based contrast agent) into separate images of bone and vasculature. However, a limitation associated with the use of a lead-based dual-energy contrast agent is that the high K-edge energy of lead (88 keV) necessitates the use of a high x-ray tube potential (e.g., 140 kVp) to achieve adequate spectral separation.^5^ However, many preclinical micro-CT scanners are limited to maximum tube potentials of ∼80 to 90 kVp, which reduces the effectiveness of DECT with Pb-based contrast agents (due to limited photon flux above the K-edge at 88 keV).

A DECT-compatible erbium (Er)-based *ex vivo* vascular perfusion contrast agent has been previously developed and characterized.^22^ The Er-based suspension is ideally suited for DECT as its absorption K-edge (i.e., 57.5 keV) is located close to the mean energy of micro-CT scanners with a 90-kVp maximum tube potential. Nonetheless, optimizing DECT for an Er-based agent requires spectral shaping tailored to erbium’s K-edge, through the addition of x-ray filtration. Typically, the low- and high-energy images will be acquired sequentially, with different x-ray filters; this leads to the potential for geometric misregistration between image volumes, due to nonreproducible scanner gantry movements. It may, therefore, be necessary to implement a method of image co-registration between acquired low- and high-energy image volumes.

This study outlines the design, implementation, and evaluation of optimized DECT on a preclinical cone-beam gantry-based micro-CT scanner. In this investigation, we describe a technique for the fabrication of: (1) custom x-ray filters to facilitate the needed spectral separation on preclinical high-resolution gantry-based micro-CT scanners; (2) fiducial marker-based image co-registration to correct for inherent micro-CT scanner bed and gantry movement between sequential scans; and (3) a motorized filter-exchange mechanism for automated DECT acquisition. The evaluation of DECT was visually and quantitatively confirmed through the automated decomposition of rat hindlimbs—perfused with an Er-based vascular contrast agent—into individual volumes of soft tissue, bone, and perfused vessels. The combination of the readily available techniques and materials outlined throughout this study will allow users of a large installed base of micro-CT scanners—typically limited to scanning at a single x-ray energy at a time—to perform optimized DECT.

## 2 Materials and Methods

### 2.1 Spectral Shaping and Modeling

X-ray spectra were modeled using a previously developed computational tool for x-ray spectral simulation (Spektr 2.0).^23^ This model incorporated CT scanner-specific parameters, such as target angle of 15 deg, source-to-isocenter distance of 39.84 cm, source-to-detector distance of 45.19 cm, additional anode inherent filtration equivalent to 1.6 mm Al (Dunlee, DU 404), and 2 cm Lexan. Spectra were modeled with varying thicknesses of x-ray filtering materials and simulated at 0.5-kVp increments. All modeling and calculations were performed within MATLAB (R2016b, MathWorks Inc., Natick, Massachusetts, USA).

Selection of the optimal parameters for low- and high-energy DECT scans involves the choice of x-ray energy (i.e., kVp), type of filtration (i.e., elemental composition), and thickness of filtration. The process necessarily involves a balance between optimizing x-ray photon flux while maintaining sufficient spectral separation. Added filtration is used to increase the mean energy of the spectrum and to reduce the width of the spectrum. In the absence of filtration (i.e., maximum photon flux), high signal-to-noise ratio (SNR) images can be acquired; however, the lack of spectral separation between the low- and high-energy spectra will reduce the accuracy of DECT decompositions—visualized as misclassified voxels between decomposed volumes. Conversely, excessive filtration will enhance spectral separation, yet the resulting diminished photon flux will result in poor SNR images, again compromising DECT decomposition accuracy.

We chose 90 kVp as the tube potential for the high-energy spectrum, as the mean energy of the unfiltered spectrum (∼42.7  kVp) is close to the Er’s absorption K-edge (57.5 keV). Additionally, 90 kVp is typically the maximum tube potential of a large installed base of laboratory micro-CT scanners. The low-energy spectrum was set at 70 kVp, to provide efficient x-ray production and ensure adequate photon flux just below the absorption K-edge of Er. With the low- and high-energy tube potentials selected, it was necessary to choose materials for differential x-ray filtration to facilitate the spectral separation required for optimized DECT.

**Fig. 1:**
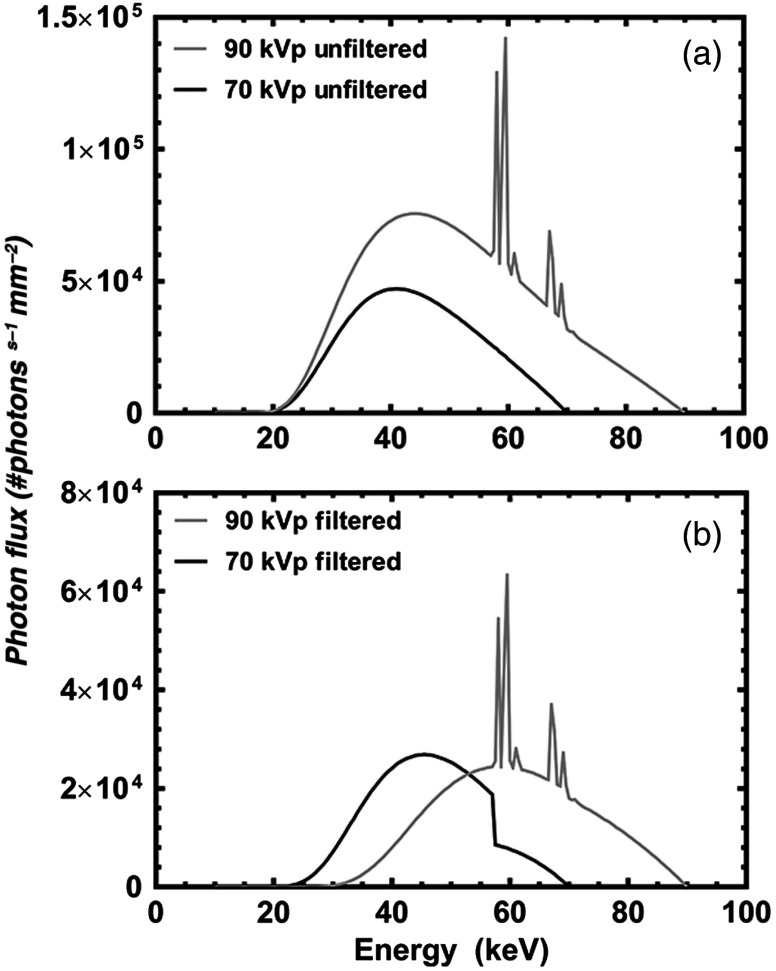
Computer modeled spectral distributions of the chosen 70- and 90-kVp low- and high-energy spectra, respectively. (a) Results of the modeled unfiltered 70- and 90-kVp spectra. (b) Modeled spectra of 70 and 90 kVp with the addition of filtration to increase spectral separation and reduction of overall photon flux.

To filter the high-energy spectrum, copper (Cu) was selected to preferentially attenuate low-energy photons, thereby shifting the mean energy higher. In addition, copper is available in foil-form at varying thicknesses and low cost; it is widely used as an x-ray filter for both single-^24^^–^^26^ and dual-energy CT.^5^^,^^27^ To model the behavior of Cu, the x-ray cross section of Cu was obtained from the National Institute of Standards and Technology’s (NIST) online database. Using the modeled output x-ray spectrum of our micro-CT scanner (GE Vision 120, GE HealthCare, London, Ontario), a total Cu path length of 550  μm was calculated for a photon flux reduction of 70% [Fig. 1(b)]. Previous research with a rat hindlimb perfused with a Pb-based agent^5^ has shown that a 70% photon flux reduction (i.e., 30% photon transmission) resulted in sufficient spectral separation to facilitate the decomposition of DECT images into individually segmented bone and perfused vessel images.

To filter the low-energy spectrum, we selected Er, as it will inherently attenuate photons above 57.5 keV. Using the cross-sectional values of Er from NIST, a calculated Er thickness of 68  μm would provide a 50% photon flux reduction, generating a photon flux similar to that of the filtered high-energy spectrum [Fig. 1(b)].

To experimentally examine the spectral separation resulting from the addition of our proposed custom x-ray filtration (i.e., Er and Cu filters for the low- and high-energy scans, respectively), the half-value layer (HVL) of aluminum (Al) was measured *via* an Al step wedge scanned at both 70 and 90 kVp in the absence and presence of a fabricated (as described below) Er or Cu filter, respectively. The addition of x-ray filtration will preferentially absorb low-energy photons, resulting in a hardened beam with higher mean x-ray energy. Thus, increases to Al thickness (i.e., increased HVL) are required to achieve a 50% reduction in photon flux. The HVL for each energy and filtered case was calculated by plotting $\ln(\frac{I}{I_{0}})$ as a function of Al thickness, where the slope of the line represented the attenuation coefficient μ. Using μ, we then can calculate the required Al thickness for $\ln(\frac{\frac{I_{0}}{2}}{I_{0}})$ [i.e., ln(0.5) or a HVL].

### 2.2 X-Ray Filter Fabrication

The Cu and Er filters described above can be implemented on bench-top specimen micro-CT scanners by placing metal foils at the x-ray tube port, prior to the sample. However, for use with gantry-based micro-CT scanners, it may not be possible or practical to modify the system in this manner. The addition of a mechanism to mount the filters on the tube port—and exchange them between scans—may interfere with the normal operation of the scanner and gantry balance. For these reasons, we elected to implement an annular cylindrical filter that surrounds the scan bed yet fits within the scanner bore. This approach to filtration avoids modifications to the scanner and is compatible with gantry-based scanners.^5^ Although the annular filter acts through a combination of pre- and postobject filtration, it provides a total attenuation that is equivalent to a preobject filter of identical path length.

A high-energy Cu x-ray filter was fabricated from readily available Cu foil. Sheets of 0.08-mm Cu foil were wrapped around an acrylic annular cylinder, with dimensions of 8.2-cm outer diameter (OD) × 6.4-cm height (H) × 0.3-cm wall thickness (WT). These dimensions were chosen to be slightly less than the maximum field-of-view (FOV) size for the selected micro-CT scanner (Vision 120, GE HealthCare, London, Ontario, Canada), facilitating the reconstruction of the entire x-ray filter and sample during DECT-acquisitions. A total of three individual layers of Cu foil provided a total path length of 0.48 mm, resulting in a photon flux reduction of 66%. However, unlike the high-energy x-ray filter, fabrication of the low-energy Er x-ray filter remained challenging, as sufficiently large sheets of Er are not readily available and may be prohibitively costly. Therefore, this led us to develop a new methodology, which allowed the creation of cast nanopowder-incorporated resin-based annular filters; in our case, inexpensive erbium oxide nanoparticles.

**Fig. 2:**
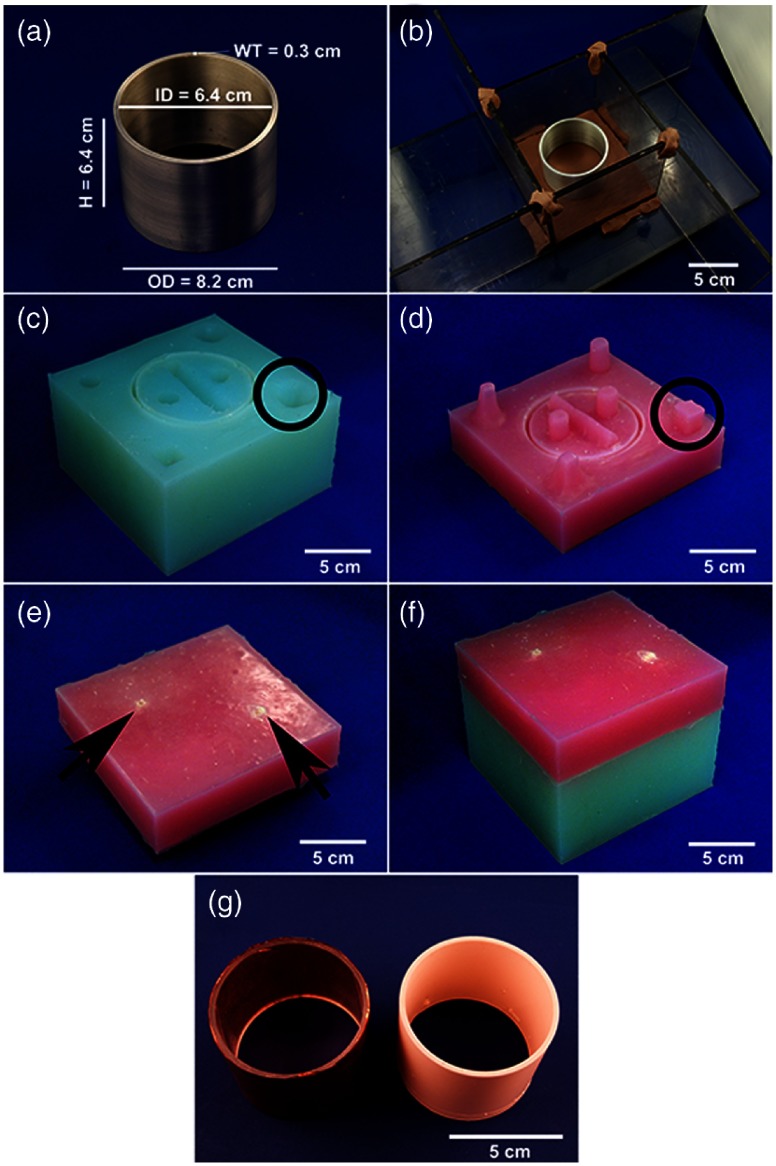
Process implemented to fabricate a custom silicone mold used to cast custom resin x-ray filters. (a) Master machined Al filter. (b) Assembled box, comprised of multiple acrylic pieces, to encompass the two-part silicone that is to be poured over the embedded Al filter within a layer of sulfur-free clay. (c) Extracted silicone mold representing the bottom-half of the silicone mold. Circle emphasizes one of the seven registration keys that were used to ensure accurate assembling of the silicone bottom- and top-half. (d) Silicone mold of the top-half. Circle represents the corresponding registration key from (c). (e) Top-half of the silicone mold flipped to demonstrate the fill and evacuation ports (arrows). (f) Assembled silicone mold. (g) Fabricated custom x-ray filters, the erbium-impregnated resin casted low-energy filter (right) and the copper foil wrapped around acrylic core high-energy filter (left).

To craft a custom cylindrical Er x-ray filter, a master filter shape and its respective negative silicone mold were required. The master cylindrical annular shape was machined from a solid cylindrical Al stock (Al, Alloy 6061, McMaster-Carr, Aurora, Ohio, USA) until dimensions of 8.2-cm OD × 6.4-cm H × 0.3-cm WT were achieved. The bottom-half of the mold was constructed by embedding the Al filter in a thin layer (∼1  cm) of sulfur-free clay (Monster Clay, Ohio, USA) within an acrylic “box” [Fig. 2(b)]; exact box dimensions are not critical. Several registration keys, fashioned from clay, were placed throughout the mold. The silicone elastomer mixture was prepared as instructed by the manufacturer (Bluestar Silicones RTV 4420 QC, New Jersey, USA), poured into the acrylic box and allowed to cure [∼1  h, Fig. 2(d)]. The respective top-half of the mold was created by inverting the cured silicone and Al filter, removing the clay and spraying exposed surfaces with mold release (Smooth-Cast^®^ Ease-Release, Pennsylvania, USA). Fill and evacuation ports were modeled from clay and affixed to the Al filter. A second batch of silicone elastomer mixture was poured to cover the exposed Al filter and allowed to cure [Figs. 2(d) and 2(e)]. The tight fit of the co-registered two-part mold [Fig. 2(f)] would prevent leaking of the poured Er-infused resin casting mixture.

To cast the low-energy Er x-ray filter, unprocessed erbium oxide (${\mathrm{Er}}_{2}O_{3}$, American Elements, California, USA) nanoparticles (nominal diameter ∼50  nm) were incorporated within a 9-min pot-life two-part resin mixture (Smooth-Cast^®^ 321, Smooth-On, Pennsylvania, USA). To create an effective Er-foil path length of 68  μm, 5.27 g of ${\mathrm{Er}}_{2}O_{3}$ was mixed thoroughly with 30 mL of Part B and placed in a vacuum chamber (∼101.3  kPa) until all air bubbles had been evacuated. An equal volume of part A (30 mL) was carefully mixed with the part B and ${\mathrm{Er}}_{2}O_{3}$ mixture for a total of 2.5 min. The resin mixture was placed back into the vacuum chamber for an additional 2.5 min (achieving ∼68  kPa). To minimize introduction of air bubbles during casting, the resin mixture was poured as a slow and thin continuous stream into the fill port of the silicone mold [Fig. 2(f)]. The Er-infused resin was cured overnight before extraction and removal of excess resin [Fig. 2(g)]. The cast low-energy Er x-ray filter resulted in a 49% reduction of photon flux.

### 2.3 Dual-Energy Microcomputed Tomography

All samples were scanned with our DECT protocols on a preclinical gantry-based cone-beam micro-CT scanner (Vision 120, GE Healthcare, London, Ontario, Canada). The low-energy scan parameters were 70 kVp, the additional Er-cast resin filter (as previously described), and 50 mA. The high-energy was acquired at 90 kVp, with the previously mentioned Cu filter, and 40 mA. Both low- and high-energy scans were acquired with 50  μm isotropic voxel spacing, 1200 projections at 0.3-deg increments over 360 deg, 10 frames averaged per projection, and 16 ms per frame. The total time required for each energy scan was ∼1.5  h, which included the time required for gantry motion and recording of image projections; thus a complete DECT scan was ∼3  h. Reconstructed three-dimensional (3-D) images were rebinned 2×2, resulting in 100-μm isotropic voxel spacing. Images were rescaled into Hounsfield units (HU) using vials of water and air within the FOV.

#### 2.3.1 Image co-registration

**Fig. 3:**
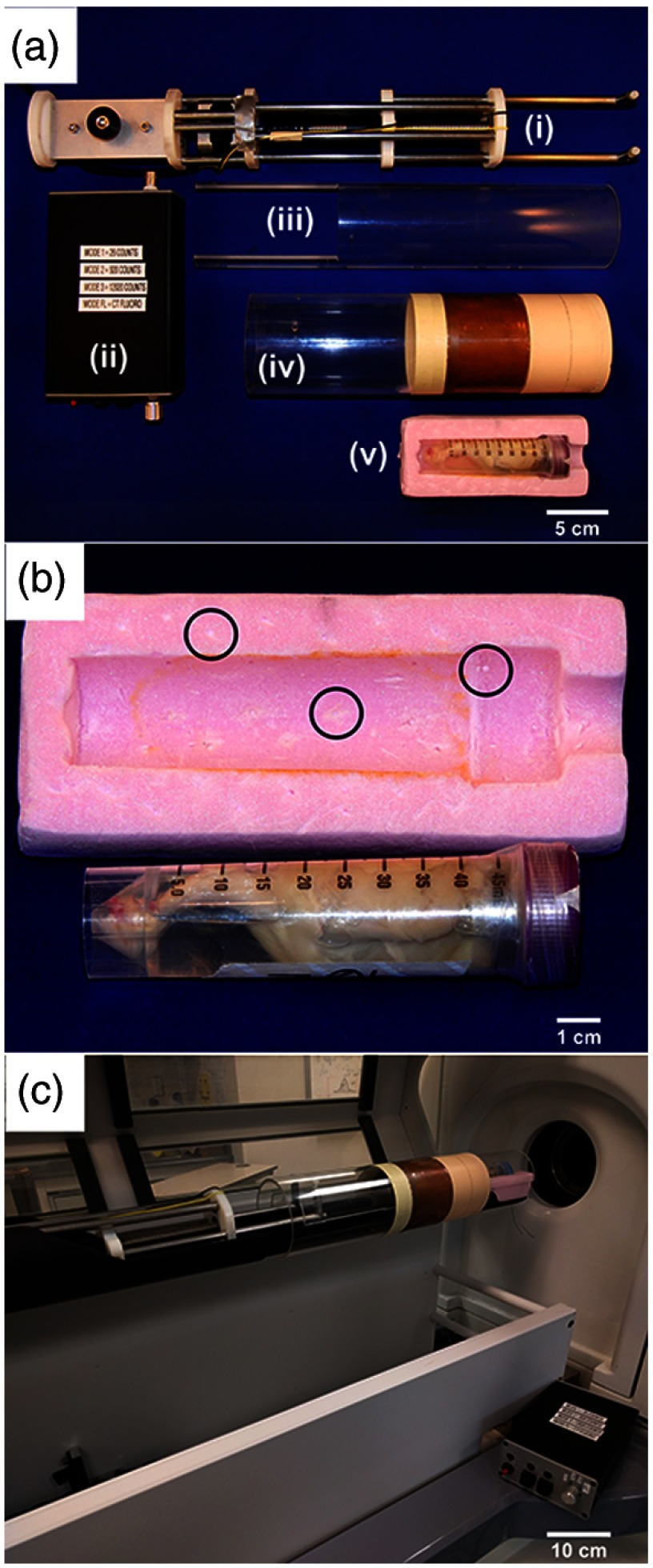
Instrumentation implemented on the micro-CT scanner, which facilitated the switching of x-ray filters and aided in image co-registration. (a) Individual pieces of the automated filter-exchange mechanism: (i) linear actuator filter-switcher; (ii) control box for filter-exchange mechanism; (iii) clam shell which resides on the CT scan bed to support the x-ray filters; (iv) custom Er and Cu x-ray filters mounted to an acrylic cylinder; and, (v) radiolucent sample holder and sample. (b) Enhanced view of sample holder (a-v) to emphasize the many embedded fiducial markers, three of which have been encircled. (c) The entire setup assembled on our micro-CT scanner.

Fiducial markers beads [1.6-mm polytetrafluoroethylene (PTFE), Teflon^™^, Fig. 3(b) circles] were embedded in a distributed pattern [Figs. 3(a) and 3(b)] throughout a custom radiolucent polystyrene foam sample holder. Fiducial markers of PTFE were chosen as they provided sufficient contrast (making them easily segmented) and introduced minimal image artifacts. The centroid of a minimum of eight fiducial markers were used to derive a transformation matrix using a least-square fitting method;^28^ wherein the high-energy image was rigidly transformed to the low-energy image with subvoxel accuracy.

#### 2.3.2 DECT automation

To increase sample throughput and reduce operator dependencies, a motorized filter-exchange mechanism (Fig. 3) was constructed to automate the DECT acquisition process. The filter-exchange mechanism automatically switched x-ray filters within the scanner bore, in between the low- and high-energy scans. Mounted on an acrylic cylinder (with an OD and WT identical to the master Al filter), the custom Er and Cu x-ray filters were actuated with a motor-driven lead screw [Fig. 3(a)(i)] controlled by an embedded microcontroller [Arduino Uno, Fig. 3(a)(ii)]. The filter-exchange mechanism assembly was secured on the micro-CT scan bed with a simple screw-jack system.

### 2.4 Contrast Agent Preparation

The preparation of the Er-based *ex vivo* vascular perfusion contrast agent has been previously described by Tse et al.;^22^ briefly, perfusion of a sample required the formulation of a catalyzing curing agent and the Er-based suspension.

The curing agent and Er-based contrast agent were prepared prior to perfusion in accordance to Tse et al. The curing agent was comprised of 60% v/v dibutyl tin dilaurate and 40% v/v tetraethylorthosilicate mixed until a homogeneous transparent pale-yellow color was achieved. The Er-based contrast agent was comprised of a two-part silicone elastomer (Microfil 132, FlowTech Inc., Massachusetts, USA) with uniformly dispersed ${\mathrm{Er}}_{2}O_{3}$ nanoparticles (nominal diameter 50 nm, Nanostructured and Amorphous Materials, Texas, USA). To create a volume of 30 mL, sufficient for a single rat hindlimb perfusion, 4.0 g of ${\mathrm{Er}}_{2}O_{3}$ (13.3% w/v) was mixed within 8.73 mL of MV-132 and 17.47 mL of MV-diluent and probe sonicated for a total of 35 min.^22^ The remaining 3.8 mL was comprised of the curing agent, as previously described, added prior to perfusion.

### 2.5 Rat Hindlimb Perfusion

A custom catheter with sufficient flexibility was created to aid in its manipulation during surgery and prevention of accidental vessel tearing upon insertion. The catheter was comprised of a blunted 18 G (BD, New Jersey, USA) needle with 15 cm of polyethylene tubing (#1417011F, Fisher Scientific, New Hampshire, USA) and 10-cm silicone tubing tip (#60985-724, VWR, Pennsylvania, USA). To join the silicone and polyethylene tubing, the silicone tubing was placed in diethyl ether (Sigma Aldrich, Michigan, USA) for ∼10  s, causing the tubing to swell and ease its placement over the polyethylene tubing. A bevel was introduced on the tip of the silicone tubing.

The Animal Use Subcommittee at the University of Western Ontario approved all animal experiments. Ten male wild-type Sprague Dawley rats (Harlan, Indianapolis, Indiana, USA) were anesthetized and maintained with 3% isoflurane (in 2% $O_{2}$) (Sandoz, QC, Canada). Five minutes prior to surgery, a jugular injection of 500  μL heparin (to prevent blot clotting) was administered. An incision along the abdomen was made, and organs were parted till the aorta and inferior vena cava (IVC) were visualized. The parietal peritoneum covering the IVC and aorta was carefully separated from the underlying vessels using gauze. Two lengths of silk thread (∼8  cm) were passed in between the separated aorta and IVC. One length of thread was used to tie off the aorta below the renal artery. Downstream of the tied-off aorta, a small incision was made in the aorta. The custom catheter (as previously described) was inserted and maneuvered until the tip of the catheter was ∼1 to 2 cm above the aortic bifurcation. The second thread was gently tied off around the aorta and catheter, holding the catheter in place. The IVC was severed to allow for circulatory drainage. Hindlimbs were flushed with ∼250  mL of 0.4% heparinized saline prior to perfusion of the Er-based contrast agent.

3.8 mL of the prepared curing agent was added to the Er-suspension and vortexed continuously for 8 min. The mixture was injected into an IV bag and suspended 69 in. above the animal, equivalent to 129 mm Hg. The contrast agent was perfused until cured (i.e., ∼37  min postaddition of curing agent). Rat hindlimbs were fixed in 10% formalin for at least 2 weeks prior to the excision of the left hindlimb, its embedding within agar, and scanning with the previously outlined DECT protocols.

### 2.6 Image Processing

To assess the homogeneity (i.e., uniform distribution of ${\mathrm{Er}}_{2}O_{3}$ nanoparticles) of the cast Er-embedded resin filter, the entire Er-filter was scanned at 90 kVp, 40 mA, 900 views, 0.4-deg increments over 360 deg, 16-ms exposure, and total scan time of 5 min. The resulting volume was rebinned 2×2 for a final resolution of 100  μm. The mean CT values from 10 randomly placed 300×300×300  μm regions-of-interest (ROIs) (MicroView, v2.2.RC5, GE HealthCare, London, Ontario, Canada) throughout the scanned Er-filter were recorded and analyzed with a t-test, and significance was achieved if p<0.05.

Decomposition of all presented DECT images were performed *via* matrix factorization, as previously outlined by Granton et al.,^5^ and a more detailed explanation has been included in the attached Appendix. However, briefly, the decomposition required six values, represented by the CT intensity values (determined through an iterative approach) of soft tissue, bone, and vessel from both low- and high-energy scans. These values were obtained from ROIs within the bicep femoris region (soft tissue), cortical bone (bone), and the femoral artery (vessel). The generated decomposed volumes represented quantitative maps of each individual component, with voxel values (0 to 10,000) representing the volume fraction (0% to 100%, respectively) or the percent contribution of the decomposed material within each individual voxel; the remaining percentages were comprised of a mixture of the two remaining components.

Quantitative evaluation on the accuracy of DECT decomposition accuracies was performed on the perfused 10 rats by quantifying the number and distribution of misclassified voxels within each decomposed volume (i.e., soft tissue, bone, and vessel). To quantify misclassified voxels, the coordinates of ROIs (500×500×500  μm, MicroView) within areas of soft tissue (bicep femoris), bone (cortical bone), and vessel (femoral artery) were recorded. Although the chosen ROI locations were from the bicep femoris, cortical bone, and femoral artery, they were not identical to the ROI locations chosen for the determination of the six values, as previously mentioned, required for DECT decomposition. Thus, for each rat, the three recorded ROIs were transposed within each decomposed volume of soft tissue, bone, and vessel. For each decomposed component, the recorded mean values from the 10 samples were averaged. The sum of each tissue ROI across all decomposed volumes will equal 10,000 arbitrary units or 100% (e.g., the sum of the mean values from femoral artery ROI transposed into the soft tissue, bone, and vessel decomposed volume will be 10,000 arbitrary units). Therefore, the normalized recorded values from all 10 samples provided the percent of voxels that have been misclassified as another tissue.

All statistical analysis was performed using Prism (GraphPad, v7.03, La Jolla, California, USA). A statistical significance was achieved if p<0.05.

The use of 3-D visualization software (VGStudio Max 2.0, Heidelberg, Germany) provided further visual enhancements, such as colorization and visual interactions between individual components.

## 3 Results and Discussion

### 3.1 DECT Design and Implementation on a Preclinical Micro-CT Scanner

In this study, we have designed and implemented custom x-ray filtration, an automated filter-exchange mechanism, and fiducial marker-based image co-registration to successfully decompose—with high accuracy—DECT-acquired images from a preclinical gantry-based cone-beam micro-CT scanner.

Using simple silicone casting techniques, we created a silicone mold that facilitated the fabrication of an inexpensive and homogenous custom annular cylindrical Er-impregnated resin x-ray filter [Fig. 2(g)]. Excluding material costs to produce the silicone mold (∼$50 for the stock Al and silicone), the cost of materials to cast a single Er x-ray filter was ∼$6, significantly cheaper than any Er-foil counterpart. The homogeneity test revealed a statistical significant, but vanishingly small (given the noise of ± 60 HU), difference of p<0.001. However, the overall mean and standard deviation of 2753±29 HU suggested a homogeneously cast Er x-ray filter.

The constructed motorized filter-exchange mechanism [Figs. 3(a) and 3(c)] successfully automated the acquisition of DECT images. This eliminated the need for operator-dependent filter switches and possible operator errors (i.e., incorrect filter choice and inadvertent sample motion).

Easily segmented fiducial markers [Fig. 3(b)] aided the semiautonomous, subvoxel image co-registration, where operator intervention was required only to choose a seed-point for the automated centroid calculation and co-registration. Together, the automated DECT acquisition and image co-registration provided a nearly automated work-flow for accurate DECT decompositions (as shown below).

### 3.2 DECT Results

Increases to the Al HVL thicknesses were observed in both 70- and 90-kVp scans upon the addition of our Er-based resin filter and Cu filter, respectively. At 70 kVp, the HVL increased from 4.45 to 5.35 mm. For 90 kVp, the HVL thickness increased more dramatically from 5.96 to 8.83 mm. The greater change in HVL thickness within the high-energy case (8.83−5.96=2.87  mm) when compared to the low-energy case (5.35−4.45=0.9  mm) confirmed that the implementation of our Er-based resin and Cu x-ray filters resulted in increased spectral separation.

**Fig. 4:**
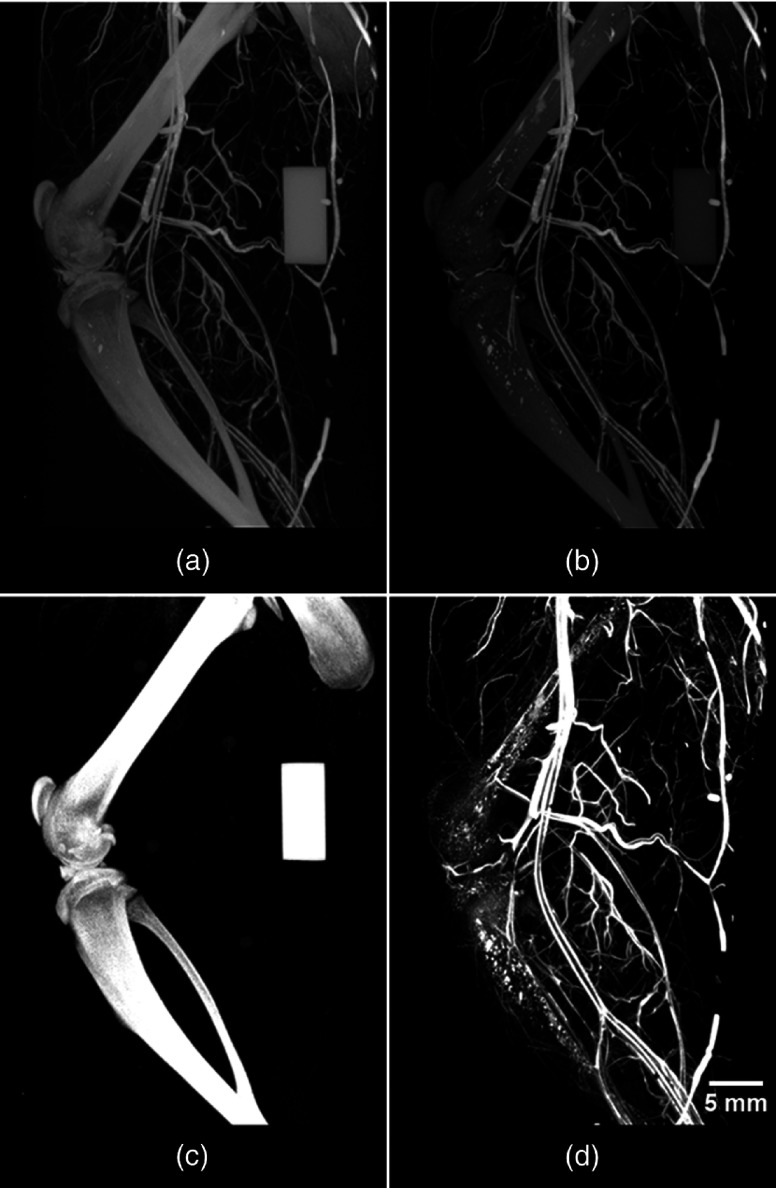
DECT results of an Er-perfused rat hindlimb. Displayed are the low- and high-energy images acquired with the previously outlined DECT protocols and implemented automated filter-exchange mechanism and custom x-ray filtration. The acquired (a) low- and (b) high-energy images are decomposed automatically into their respective (c) bone- and (d) vessel-only components. The accurate decomposition of bone and vessels facilitated the visualization of vessels within the cortical bone, in addition to the vascularized sheets lining the outside and inside of each long bone, periosteum, and endosteum, respectively. The ability to visualize these vessels manifests as femur- and tibia-like structures in the vessel-only image. Note the absence of the bone-mimicking calibrator from the vasculature image, emphasizing the success of the DECT decomposition.

Following an iterative approach, as outlined in the included Appendix, the six values required for DECT decomposition (i.e., CT intensity values of soft tissue, bone, and contrast-enhanced vessel at low and high energy) are summarized in Table 1. Implementing the previously described matrix factorization,^5^ these six values facilitated the DECT decomposition of an Er-perfused rat hindlimb into separate and quantitative 3-D volumes of soft-tissue, bone [Fig. 4(c)], and perfused vasculature [Fig. 4(d)].

**Table 1:** The representative six numbers (i.e., CT intensity values of soft tissue, bone, and contrast-enhanced vessels at both low and high energy) chosen as the input parameters for the automated decomposition algorithms. The six numbers were acquired from an iterative approach outlined within the included Appendix.

	Soft tissue	Bone	Contrast-enhanced vasculature
Low energy	37	2743	1659
High energy	0	1909	2059

**Fig. 5:**
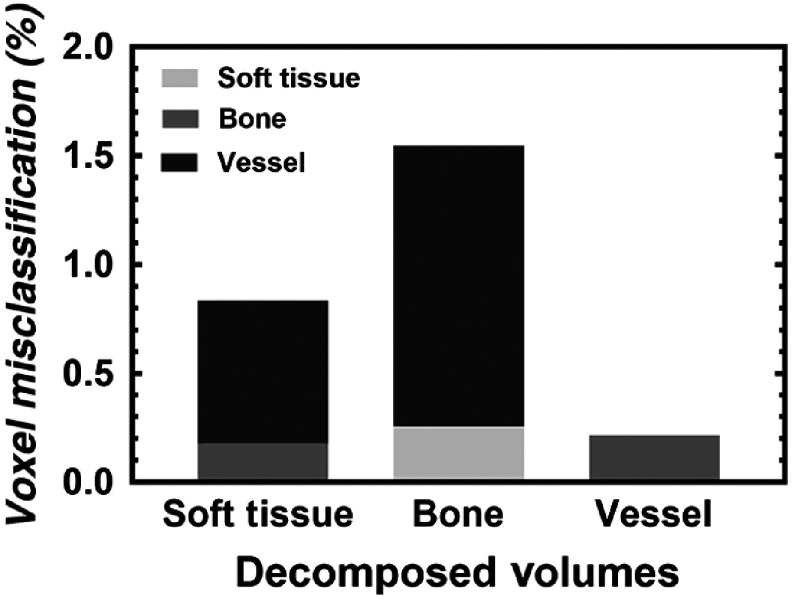
Graph depicting the percent and composition of misclassified voxels, from Table 2, after automatic DECT decomposition. Within each decomposed volume, misclassified voxels are comprised of the remaining two components.

The DECT decomposition yielded accuracies of 99.18%, 98.45%, and 99.78% in the soft tissue, bone, and vessel volumes, respectively (Table 2), as quantified from 10 perfused rats. The visual representation of the amount and composition of misclassified voxels within each decomposed volume is shown within Fig. 5.

**Table 2:** DECT decomposition quantitative assessment. 500×500×500  μm ROIs were placed within known areas of soft tissue, bone, and vessels in each decomposed volume. The mean values of each ROI were recorded and normalized to 100% within each individually decomposed volume.

		Known pure components		
		Soft tissue	Bone	Vessel
Decomposed volumes	Soft tissue	99.18 ± 0.44	0.26 ± 0.64	0.01 ± 0.03
	Bone	0.18 ± 0.10	98.45 ± 1.40	0.20 ± 0.38
	Vessel	0.66 ± 0.45	1.29 ± 1.03	99.78 ± 0.42

**Fig. 6:**
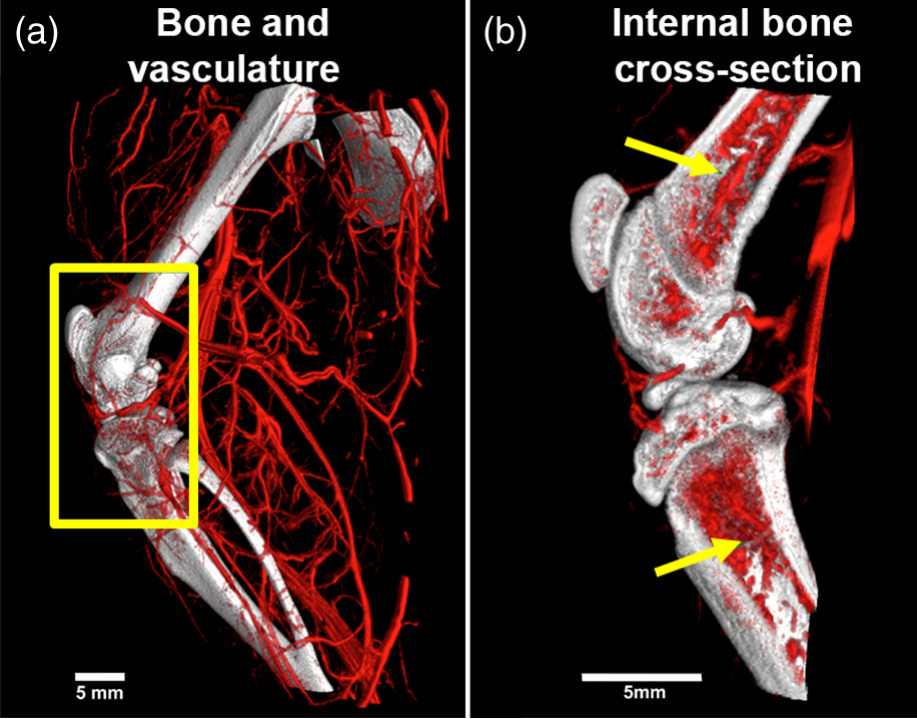
DECT results after processed with 3-D visualization software to emphasize the interactions between decomposed components—vessels (red) and bone (white). (a) Overall view of the vasculature outside and on the surface of the perfused rat hindlimb. (b) An internal cross-sectional view of the area outlined in yellow from (a). Note the vascularized internal nature of bone. Yellow arrows denote the primary nutrient vessels of the femur and tibia.

Isolated visualization of the soft tissue, bone, and vessels is achieved *via* DECT decompositions. To image interactions between individual components, the decomposed data can be processed with 3-D visualization software. In our case, the addition of color to vessels (red) and bone (white) provided enhanced visualization to emphasize the vasculature surrounding and traversing within bone (Fig. 6).

### 3.3 Importance of X-Ray Filtration and Image Co-Registration

Integration and implementation of multiple techniques (i.e., custom x-ray filtration, automated filter-exchange mechanism, fiducial markers, and image co-registration) are required for the optimal performance of DECT on preclinical gantry-based cone-beam micro-CT scanners. Any deficiency or absence in any of these techniques (i.e., spectral shaping and image co-registration) will result in nonideal DECT decompositions. We demonstrated the importance of spectral shaping (*via* x-ray filtration) and image co-registration by performing the following experiments and data reanalysis.

**Fig. 7:**
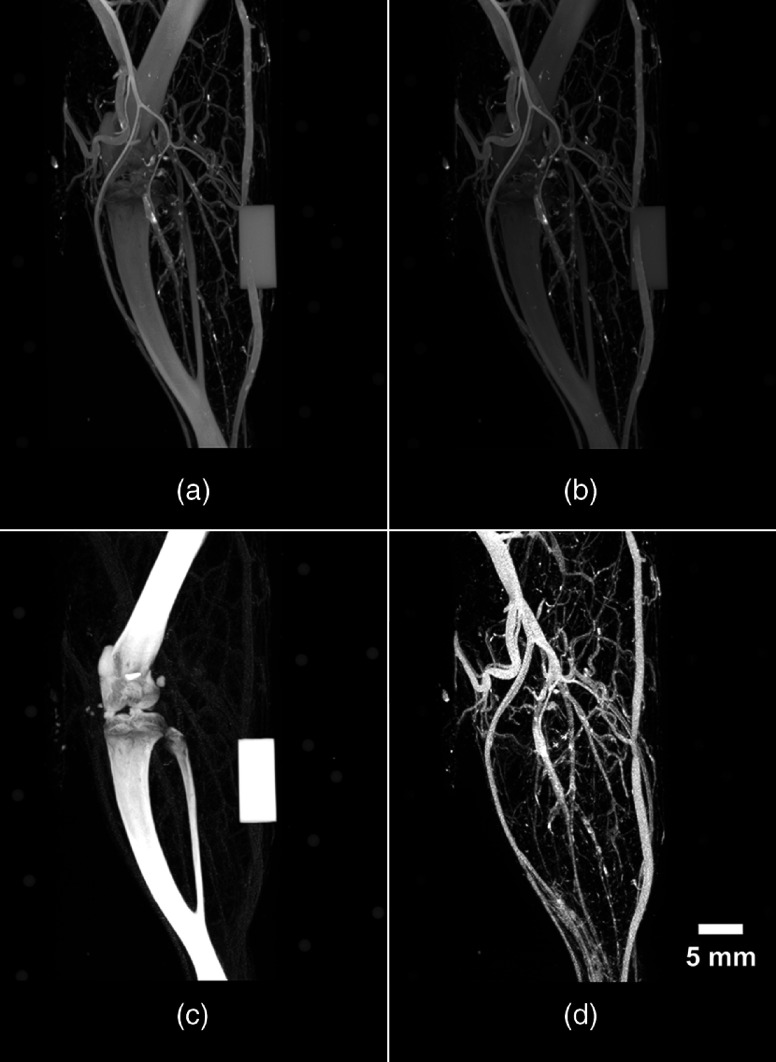
DECT results of the Er-perfused rat hindlimb if no spectral shaping was implemented during the collection of the dual-energy images. Similar to results presented in Fig. 4, DECT-acquired (a) low- and (b) high-energy images were acquired with the previously outlined DECT protocol, in the absence of the fabricated custom low- and high-energy x-ray filtration, co-registered images, and a separate set of six values (as without x-ray filtration, CT values of pure soft tissue, bone, and vessel will be different than in the presence of x-ray filtration) and were utilized for decomposition. Results of the decomposition are displayed in (c) bone- and (d) vessel-only image. Note the misclassified vessel voxels (i.e., “bleeding”) within the bone image.

**Fig. 8:**
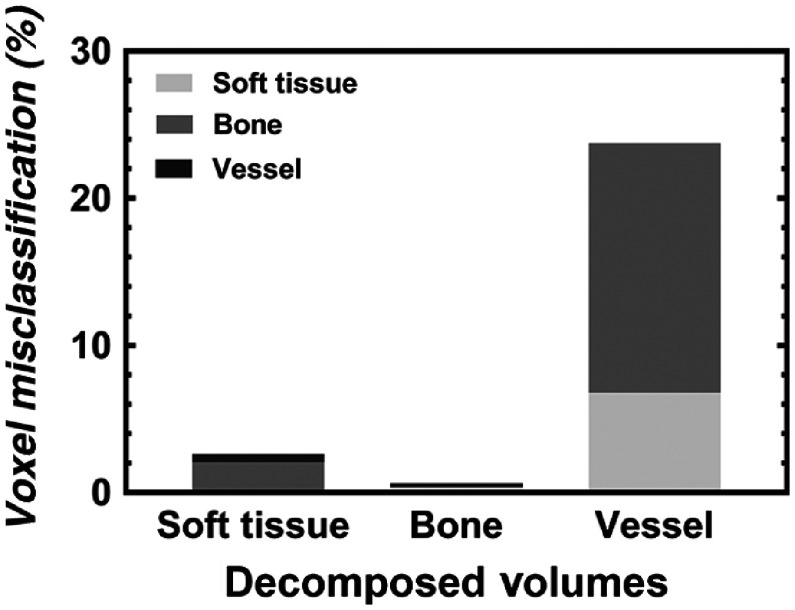
Graph depicting the percent of misclassified voxels, from Table 3, after automatic DECT decomposition. Within each decomposed volume, misclassified voxels are comprised of the remaining two components.

Five Er-perfused rat hindlimbs were rescanned (with the previously outlined DECT protocols) in the absence of customized x-ray filtration. The resulting suboptimal spectral shaping and large spectral overlap are visualized in Fig. 1(a). Acquired low- and high-energy scans were co-registered and decomposed with a new set of six CT intensity values (determined through the iterative approach outlined within the Appendix): low-energy soft tissue (45), bone (2930), vessel (2647), and high-energy soft tissue (45), bone (2572), and vessel (2768). The DECT decomposition resulted in a large number of misclassified voxels between decomposed volumes (Fig. 7), visualized as “bleeding” between components. Visually, the perfused vasculature [Fig. 7(d)] appears to have been decomposed properly; however, quantitative analysis revealed that only 76.23% of the vessels had been classified correctly (Table 3), with the majority misclassified as bone [Fig. 7(b)]. The remaining percentages (23.77%) and compositions of misclassified voxels are presented in Table 3 and visualized within Fig. 8.

**Table 3:** DECT decomposition quantitative assessment in the absence of proper x-ray filtration. 500×500×500  μm ROIs were placed within known areas of soft tissue, bone, and vessels in each decomposed volume. The mean values of each ROI were recorded and normalized to 100% within each individually decomposed volume.

		Known pure components		
		Soft tissue	Bone	Vessel
Decomposed volumes	Soft tissue	97.33 ± 0.54	0.36 ± 0.42	6.79 ± 10.53
	Bone	2.08 ± 0.81	99.37± 0.59	16.96 ± 9.66
	Vessel	0.54 ± 0.61	0.27 ± 0.59	76.23 ± 12.61

**Fig. 9:**
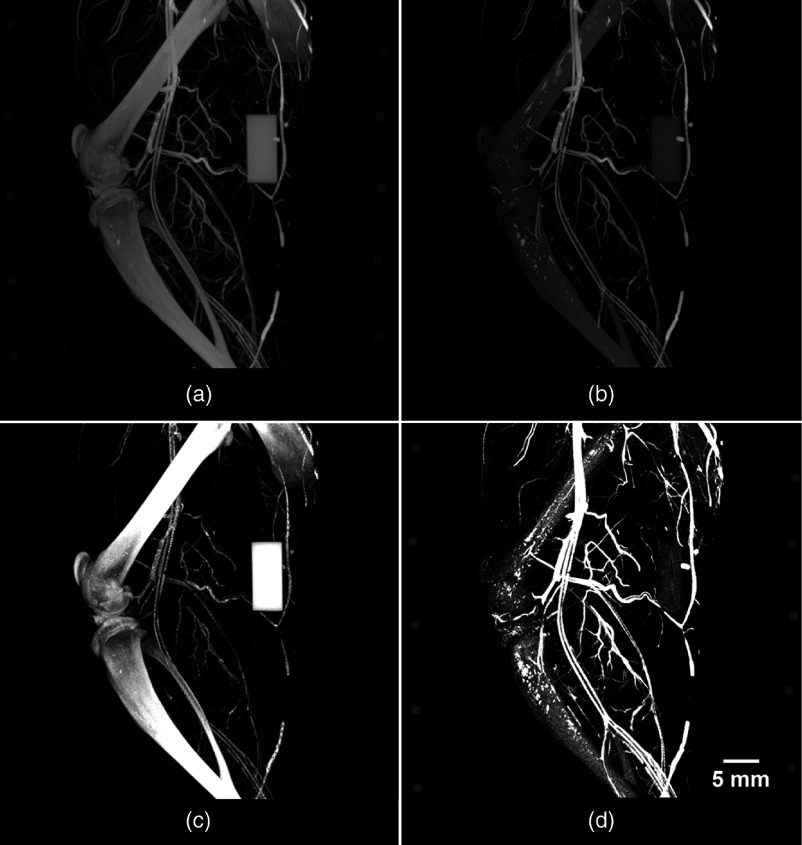
DECT results of the same Er-perfused rat hindlimb if the low- and high-energy images were not co-registered prior to decomposition. Similar to results presented in Fig. 4, DECT-acquired (a) low- and (b) high-energy images were acquired with the previously outlined DECT protocols and custom x-ray filtration; however, the fiducial markers (present on the periphery of each image) were not utilized for image co-registration. Decomposition with these images resulted in the displayed (c) bone- and (d) vessel-only image. Misregistration results in the misclassification of objects’ nonoverlapping boundaries. In the maximum intensity projections (MIP) presented here, misclassification of the boundaries of vessels and bones is emphasized by the MIP procedure. Note the large amount of “bleeding” of bone (and bone mimicking calibrator) within the vessel image and vice versa.

To establish the importance of image co-registration, data collected for Fig. 4 were reanalyzed without co-registration prior to DECT decomposition. The decomposition results (Fig. 9) appeared similar (i.e., misclassified voxels “bleeding”) to those that have been acquired without proper spectral separation (Fig. 7); however, the “bleeding” was more apparent across all decomposed volumes. Clearly, the sequentially acquired low- and high-energy volume images are not inherently co-registered at the subvoxel level; this is likely due to a combination of small (and unavoidable) variations in positioning of the scanner bed and gantry between scans.

Thus it is apparent both visually (Figs. 7 and 9) and quantitatively (Table 3) that both spectral separation and image co-registration are necessary for the proper collection and optimal decomposition of DECT-acquired images.

### 3.4 Limitations

Although our work has overcome the challenges of spectral separation and image co-registration,—required for the optimal implementation of DECT on preclinical cone-beam micro-CT scanners—several optimizations and limitations remain. The addition of our customized x-ray filtration resulted in >98% decomposition (Table 2 and Fig. 5); however, scan times of ∼3  h were required. It is possible that further optimization of the added filtration (i.e., reduction in effective filter thickness) may allow for reduced scan time, while maintaining adequate spectral separation.

A limitation within our study is the limited performance of DECT to a single FOV, as the constructed automated filter-exchange mechanism can only actuate the filters between two fixed positions. However, modifications to the filter-exchange mechanism to actuate the sample holder or allow further travel distances of the x-ray filters would facilitate DECT of whole small animals.

An additional limitation is that our perfusion procedure precludes *in vivo* studies; however, there is currently research investigating a lanthanide-based *in vivo* vascular perfusion contrast agent.^29^ These future studies would also entail researching suitable DECT x-ray filtration compatible with the vascular contrast agent, in addition to balancing an acceptable x-ray dose for *in vivo* studies.

## 4 Conclusion

In this study, our implementation of DECT on a conventional preclinical laboratory cone-beam micro-CT scanner allowed for the automatic decomposition of Er-perfused rat hindlimbs into separate, distinct, and quantitative images of soft-tissue, bone, and perfused vasculature. This was achieved by sequential acquisition with two differential x-ray spectra and incorporating subvoxel volumetric image co-registration between scans. These scans were acquired with custom-fabricated x-ray filtration, an automated filter-exchange mechanism, and embedded fiducial markers that allowed for image co-registration using a rigid matrix transformation. The automated decomposition into specific tissue components was accurate to within 2%, facilitating quantitative analysis of specimen composition within 100-μm cubed voxels (i.e., 1 nL volume elements). The additional required hardware and software modifications did not interfere with the normal operation of a conventional commercial micro-CT scanner.

As part of this implementation of DECT, this study presented a methodology for the fabrication of custom x-ray filters, optimized for the spectral shaping associated with an Er-based contrast agent. In the future, this fabrication technique can be modified to create user-specified custom (i.e., shape, elemental composition, and concentration) resin-embedded x-ray filters of any element present in nanoparticulate powders. This range of customization would facilitate the application of DECT to take advantage of other exogenous contrast agents or endogenous contrast within the specimen.

The methodology presented here will have applications in a range of biomedical research, including the study of cardiovascular disease, respiratory conditions, cancer, and osteoarthritis. Our approach for optimal spectral shaping using customized filters is also applicable in nonbiomedical research, including earth-science applications (e.g., geological specimens and meteorite analysis) and nondestructive testing of 3-D-printed objects. Additionally, the techniques that we have described within our study are applicable to a large installed base of micro-CT scanning systems, as well as conventional multislice CT scanners for research applications.

## Appendix:

$$\mu_{\text{soft tissue}(\mathrm{low}\text{-}E)}f_{\text{soft tissue}}+\mu_{\text{bone}(\mathrm{low}\text{-}E)}f_{\text{bone}}+\mu_{\text{vessel}(\mathrm{low}\text{-}E)}f_{\text{vessel}}=\mu_{\text{low}\text{-}E}.$$ (1)

$$\mu_{\text{soft tissue}(\text{high}\text{-}E)}f_{\text{soft tissue}}+\mu_{\text{bone}(\text{high}\text{-}E)}f_{\text{bone}}+\mu_{\text{vessel}(\text{high}\text{-}E)}f_{\text{vessel}}=\mu_{\text{high}\text{-}E},$$ (2)

$$f_{\text{soft tissue}}+f_{\text{bone}}+f_{\text{vessel}}=1.$$ (3)

From Eqs. (1) and (2), the observed attenuation coefficient recorded within each individual voxel in the acquired low- ($\mu_{\text{low}\text{-}E}$) and high- ($\mu_{\text{high}\text{-}E}$) energy volumes is comprised of the fractional contribution of three materials of interest (i.e., $f_{\text{soft tissue}}$, $f_{\text{bone}}$, and $f_{\text{vessel}}$) and their respective attenuation coefficient at that acquired x-ray energy (i.e., $\mu_{\text{soft tissue}(\text{low}/\text{high}\text{-}E)}$, $\mu_{\text{bone}(\text{low}/\text{high}\text{-}E)}$, and $\mu_{\text{vessel}(\text{low}/\text{high}\text{-}E)}$). In Eq. (3), we assume that a voxel can only be comprised of the fractional contribution from either soft tissue, bone, or vessel and that the sum of their fractional contribution must equal 1 (i.e., 100%).

The μ values, within Eqs. (1) and (2), for each tissue were acquired from the mean CT value in HU from 500×500×500  μm ROIs, in both low- and high-energy acquired volumes, from areas of pure soft tissue, bone, and vessel. The mean CT value was used as they are linearly proportional to the tissue’s respective attenuation coefficient. The initial six CT values (i.e., “seed values”) inputted into Eqs. (1) and (2) are dependent on known areas of pure tissues. Using improper seed values will result in nonideal decompositions (i.e., misclassified voxels, Fig. 8 and Table 3), visualized as components bleeding into one another (Fig. 7). To optimize the six seed values,—resulting in an accurate decomposition—an iterative approach was taken.

The initial six seed values were chosen by recording the mean value from 500×500×500  μm ROIs within areas of known soft tissue (bicep femoris), bone (cortical bone), and vessel (femoral artery). Unfortunately, as Er-perfused microvessels are located throughout the bicep femoris and cortical bone, the recorded CT values may be slightly higher than expected. Thus, to further finetune the seed values and obtain more accurate CT values for nonvascularized soft tissue and bone, a mask for each tissue was generated for each decomposed volume. The separate “tissue masks” were obtained by choosing a threshold value within each decomposed volume above the value of tissues “bleeding” into that volume. A custom in-house Unix-based script would then provide a mean value of all the voxels within each decomposed volume that was above the threshold value, providing a new seed value for said tissue component. This iterative approach was performed for each tissue at both low- and high-energy until an ideal set of six seed values was obtained (Table 1), resulting in optimally decomposed volumes of soft tissue, bone, and vessel (Fig. 4). An ideal set of six values was determined once the bone mimicking calibrator (SB3) and femoral artery were decomposed to >99% accuracy within the bone- and vessel-only decomposed volumes, as the bone mimicking calibrator (SB3) and femoral artery were known tissues comprised of pure bone and perfused vessel, respectively. Therefore, all that remained from Eqs. (1)–(3) are the voxel volume fraction of soft tissue, bone, and vessel (i.e., $f_{\text{soft tissue}}$, $f_{\text{bone}}$, and $f_{\text{vessel}}$).

To determine the voxel volume fractions of each tissue, we employed a matrix factorization approach to solve a system of linear equations [i.e., Eqs. (1)–(3)]. The results of these calculations performed for each voxel within the acquired low- and high-energy volumes are individual volumes of the three materials-of-interest (i.e., soft tissue, bone, and vessel). Within each decomposed volume, grayscale values (0 to 10,000) of individual voxels represented the voxel volume fraction or percent contribution (0% to 100%) of each component of that specific voxel. Thus the decomposition algorithm resulted in three tissue-distinct 3-D volumes, where grayscale values represented the percent contribution of a tissue on a voxel-by-voxel basis.
